# Improve the Design and Implementation of Metrics From the Perspective of Complexity Science Comment on "Gaming New Zealand’s Emergency Department Target: How and Why Did It Vary Over Time and Between Organisations?"

**DOI:** 10.34172/ijhpm.2020.47

**Published:** 2020-04-04

**Authors:** Junqiao Chen

**Affiliations:** The CareVoice, Shanghai, China.

**Keywords:** Emergency Room, Policy Implementation, Gaming Behaviours, Complexity Science, New Zealand

## Abstract

From the perspective of complexity science, this commentary addresses Tenbensel and colleagues’ study, which reveals varied gaming behaviours to meet the New Zealand Emergency Department (ED) metric. Seven complexityinformed principles previously published in this Journal are applied to formulate recommendations to improve the design and implementation of metrics. (1) Acknowledge unpredictability. When designing a metric, policy-makers need to leave room for flexibility to account for unforeseen situations. When implementing a metric, they need to promote sense-making of relevant stakeholders. (2) Sense-making shall be encouraged because it is a social process to understand a metric, align values and develop a coherent strategy. Sense-making is important to (3) cope with self-organised gaming behaviours and to (4) facilitate interdependencies between ED and other departments as well as organisations. (5) We also need to attend to the relationship between senior management and frontline staff. Additionally, to address one of the methodological weaknesses in Tenbensel and colleagues’ study, (6) adaptive research approach is needed to better answer emerging questions. (7) Conflict should be harnessed productively. I hope these recommendations could limit gaming in future metrics and encourage stakeholders to view inevitable gaming as an improvement opportunity.

Tenbensel and colleagues’ paper (the lead paper) reports the varied gaming behaviours across time and place with regards to the New Zealand Emergency Department (ED) target (95% of patients being admitted, discharged or transferred from an ED within 6 hours).^1^ This commentary will attempt to explain and advise the design and implementation of such metrics from the perspective of complexity science.

This perspective is chosen because both this Journal and the lead authors have track records of complexity-informed publications (for example, see Kitson and colleagues’ paper^2^ from this Journal and Tenbensel’s other papers^3,4^). These enable me to take advantage of the audience’s familiarity with complexity to dive deep into this case study. While many previous complexity-informed discussions only provided high-level ideas, I am able to draw nuanced recommendation here thanks to the rich information from this case and the larger research that it is a part of. Since a unified complexity perspective does not exist,^5^ I will sidestep academic disputes^6^ and choose the following ‘rules of thumb’ presented by a recent paper of this Journal^7^ as my pragmatic framework.^8^

## Acknowledge Unpredictability

First, acknowledge unpredictability in the design of metric. The arrival of patients to the ED and their needs are unpredictable. There will be situations in which even a very efficient ED cannot meet the target. A good metric design should provide a ‘way out,’ otherwise ‘the only means at their disposal’^1^ of frontline staff is to game the metric. For example, in United States’ clinical quality measurement schema, flexibility in denominator is allowed for medical, patient, and system reasons to account for unforeseen situations.^9^ The technical term for this is ‘exception.’ When I volunteered in an emergency medical service in the United States, we encountered a homeless patient visiting multiple EDs in a day despite no urgent treatment was needed. Aware of the situation, one ED allowed him to overstay to meet his basic needs, including having a sandwich and fully charging his phone. This ED might fail a time-centric target but what it did actually benefited the entire system. Better metric design might allow this case to be reported as an exception (for patient reasons) and removed from the denominator. Allowing flexibility in metric design could encourage clinicians to do the right things in special circumstances.

Another way to account for unpredictability is to select core populations for whom certain timely action is non-negotiable, and to allow more flexibility for other less urgent populations and actions. An example of urgent action on a core population is the interpretation of imaging for stroke patients. In the United States, a nationally-endorsed measure requires that for stroke patients who arrive at the ED within 2 hours of symptom onset, they need to have a head computed tomography or magnetic resonance imaging scan interpreted within 45 minutes of arrival.^10^ Otherwise, it is a critical failure. This idea bears resemblance to the ED triage process.^11^ It also suggests that microscopic metrics are needed to complement the main metric. Because ‘quality’ is a multifaceted and multi-layered concept^12^ (or ‘ontologically emergent’^13^), we need multi-layered metrics, each with different ranges of flexibility to handle unpredictability. Multi-layered metrics will be summarised in another rule of thumb ‘Facilitate Interdependencies.’

Unpredictable gaming might also occur during metric implementation.^14^ The lead article reports many interwoven factors that could influence behaviours, resulting in unexpected levels of gaming across study sites.^1^ Traditionally, audit is the approach to address gaming. However, audit might not work in complex adaptive systems due to its reductionist and determinist nature.^15^ Instead, I recommend a peer-to-peer social approach, which will be elaborated in the next rule of thumb ‘Encourage Sense-making.’

## Encourage Sense-Making

It was appraised that “unlike the British experience, the call for this target was ‘bottom up,’ lifted by the passion of concerned clinicians,”^16^ so ‘the passion persists and is unlikely to tolerate gaming on its patch.’ However, the target was actually recommended by an advisory group, made up of *senior* ED clinicians and managers.^17^ Since this is not entirely ‘bottom up,’ it needs to be socialised from ‘top down’ and understood by frontline clinicians (‘sense-making’). This resembles the classical metaphor of clinical guidelines versus ‘mindlines,’ the latter of which is ‘a collective sense-making by which knowledge, both explicit and tacit, is negotiated, constructed, and internalised in routine practice.’^18^ Sense-making activities have been seen in national quality initiatives such as the Australian Health Service Safety and Quality Accreditation Scheme,^19^ which is a complexity-informed initiative that undertook hundreds of consultations with health organisations to facilitate knowledge transfer and stakeholder engagement.^20^

By sense-making, various stakeholders (not just ED clinicians) could be more aligned on the goals, necessary resources and potential consequences of a metric. The objective is to engender a culture in which participants ask questions, admit ignorance, explore paradoxes and exchange different viewpoints,^7^ such that each organisation has a coherent value stance and business strategy toward a metric. As echoed in the lead paper, such coherence was not always seen within each of its study sites.

Stakeholders should also be encouraged to brainstorm possible gaming behaviours across the healthcare sector, and to apply local expert judgement on whether a certain behaviour is gaming or not in one’s local context.^21^ There is no right or wrong judgement, no blaming or retaliation (such as auditing). The objective is to increase awareness of varying local contexts, respect local expertise, and eventually strengthen professional code of conduct by holding each other accountable for fairness. Fairness is ‘a plurality of social values, perspectives and interests.’^22^ The goal of interorganisational sense-making is not to reach consensus on fairness, but to make the underline assumptions explicit and *ex ante*. A strengthened professional conduct from increased consciousness of fairness could limit ‘wild gaming’^14^ of a metric.

In brief, policy-makers, hospitals managers and clinicians need to encourage sense-making within an organisation to create a coherent business strategy, and encourage interorganisational sense-making to help learning, reflection and fairness across the industry. The next rule of thumb further analyses this.

## Recognise Self-organisation

Self-organisation is the emergence of new pattern through the activity of microscopic units that do not have access to global patterns but follow local rules. These rules could be explicit policies or customary procedures.^23,24^

In the case of our lead paper, a new pattern (skewed distribution of ED wait times) emerged through staff who, without knowledge of the global distribution, followed customary procedures of the people around them or senior staff,^25^ to modify data entry (rounding terminal digits, for example). There was no written policy to guide staff modification. How often and to what extent modification occurred was based on a staff’s own comfort level and instinct, without assessment of modifications by peers. Each person self-organised his/her gaming behaviour without coordination. (If they had coordinated, the pattern of skewness might be different.) One staff might be comfortable with rounding 369 minutes down to 360, while another staff might only be comfortable if it is within 365 minutes, for example.

To cope with this, we again need to encourage sense-making. By making the implicit rules more explicit and re-focusing on professional conduct^26^ in the discourse, wild gaming could be reduced. What is left is the ‘inevitable,’ which should be viewed as improvement opportunity for policy-makers (how to measure what matters^27^) and healthcare providers (how to provide what matters).

## Facilitate Interdependencies

‘Interdependent work across units is germane to the ED’^28^ as ED clinicians must manage challenging constantly-changing dynamics at the boundaries of the ED and other departments and organisations. This is evident in Hospital 4 in the lead paper.

To facilitate interdependencies, there needs to be metrics and resources established towards the whole system, rather than the parts.^29^ Silos and resistance are inevitable when a systems approach was absent (eg, Hospital 3). Whole-system targets encourage generative relationships among various stakeholders and provoke creative ideas.^29^ A hypothetical global metric could require patient care to be handed over to appropriate provider within six hours from the onset of the need for emergency care. Whole systems design could unite all components in the acute care journey, including pre-hospital, ED, inpatient, and post-discharge services.

The impact of whole-system targets on gaming is unknown. Based on the ‘Motives, Opportunities and Means’ framework in the lead paper, my hypothesis is that it will not reduce the opportunities or means to game, but might lower the motives. This is because now changing one data point is less likely to have a decisive system-wide effect. To effectively game a target, it will require explicit orchestrated acts from multiple players, which will be much harder to do operationally and ethically.

If this principle is viewed in combination with the first one (‘Acknowledge unpredictability’), it is advised that metrics on three levels are needed in order to promote the best outcome: one on the overall acute care, one on the care within ED, and one on a core population and/or intervention.

## Attend to Human Relationship

The lead paper provides novel findings on how relationships between frontline staff and senior management can influence gaming. In public view and research, there is a strong lack of sympathy and attention toward managers.^30,31^ The lead paper stresses managers’ varied values and struggles over time and place. It is via their interactions with frontline staff that a policy could be implemented. I add to the lead paper that not only ‘strategic behaviours’ of managers are worth studying, also their nuanced interpersonal interactions with staff, which might be best observed by ethnography (that is how Google studied their managers^32^). Even in ‘bottom-up’ policy development, it is paramount to have managers involved to provide management’s perspectives.

## Develop Adaptive Capabilities

This rule of thumb does not really apply to ED clinicians, who are already very adaptive to bridge the gap between a target (‘work as imaged’) and what could be done.^33^ Gaming is the last resort of adaptation to meet a metric.

Adaptive capabilities are needed more for policy evaluation. Studying complex adaptive systems requires ‘theoretically grounded, methodologically pluralistic, flexible and adaptive study design.’^34^ The lead paper is part of a larger research with a rigorous mixed-method protocol.^17^ From the formulation of new research questions to the secondary data analysis, the lead paper already brings new ideas on top of the original protocol, and I am appreciative to the lead authors’ pursuit of knowledge. However, a limitation is inherent in secondary use of data, that the qualitative analysis does not augment, converge with or get embedded in the quantitative analysis, or vice versa.^35^ For example, the quantitative analysis identifies terminal digit preference bias, but the qualitative analysis is not able to explain how exactly it occurred. In the lead paper, these two methods basically answer different questions, because the data of one type was not collected after being informed by the analysis of another type. For the sake of argument, rather than calling it a mixed-method study, I will call it a study with two parts, each using different methods.

This goes back to the inflexibility of the original protocol, which does not allow future work when needed. As complexity thinkers comment: ‘Research protocols consisting entirely of pre-ordained work packages arranged around a boxes-and-arrows diagram accompanied by tight milestones and timelines may be the stuff that funding panels’ dreams are made of, but … less likely to generate meaningful findings than studies which engage pragmatically with the multiple uncertainties involved and offer a flexible and emergent approach to exploring them.’^34^ Large research projects should hold back some budget to allocate as and when it is needed in the future.^36^

## Harness Conflict Productively

The conflict between *business efficiency* and *integrity of care* in ED was well studied ethnographically.^26^ The different levels of gaming are by-products from different levels of the conflict. Such tension could never be resolved,^37^ as ‘local‐level discretion at the site of service delivery, or street‐level bureaucracy, can never be fully managed out by such rational administrative logic.’^26^ To make the best of this situation, we need to build relationship, maintain dialogue, mutually respect each profession’s norm and value, and muddle through.^7^ So to answer the lead paper’s question ‘whether or not gaming is a necessary evil … in the pursuit of improved health service,’ my response is: no, it is not a necessary evil, but a *catalyst* for improvement if handled appropriately.

In conclusion, policy-makers, hospitals managers and clinicians need to work together to improve the design and implementation of metrics. Complex systems are inherently unpredictable, so we need to allow flexibility in metric design and encourage sense-making in implementation. Within an organisation, sense-making can help to cope with self-organised gaming behaviours, to facilitate interdependencies, and to develop a coherent strategy. Interorganisational sense-making could help to promote learning, reflection and fairness across the industry. It is important to view conflict as a catalyst for improvement, and to reserve research capacity to respond to emerging questions as we muddle through.

## Acknowledgements

Comments from Ms. Joanna Wu and an anonymous reviewer are gratefully appreciated, especially during a time of COVID-19 pandemic.

## Ethical issues

Not applicable.

## Competing interests

Author declares that she has no competing interests.

## Author’s contribution

JC is the single author of the paper.
